# Favoring the hierarchical constraint in penalized survival models for randomized trials in precision medicine

**DOI:** 10.1186/s12859-023-05162-x

**Published:** 2023-03-16

**Authors:** Shaima Belhechmi, Gwénaël Le Teuff, Riccardo De Bin, Federico Rotolo, Stefan Michiels

**Affiliations:** 1 Université Paris-Saclay, CESP, INSERM U1018 Oncostat, labeled Ligue Contre le Cancer, Villejuif, France; 2 Bureau de Biostatistique et d’Epidémiologie, Gustave Roussy, Villejuif, France; 3 Department of Mathematics, University of Oslo, Oslo, Norway; 4 Biostatistics and Data Management Unit, Innate Pharma, Marseille, France

**Keywords:** Biomarker-treatment interactions, Precision medicine, Hierarchical interaction, Biomarker selection, Lasso, Cox regression

## Abstract

**Background:**

The research of biomarker-treatment interactions is commonly investigated in randomized clinical trials (RCT) for improving medicine precision. The hierarchical interaction constraint states that an interaction should only be in a model if its main effects are also in the model. However, this constraint is not guaranteed in the standard penalized statistical approaches. We aimed to find a compromise for high-dimensional data between the need for sparse model selection and the need for the hierarchical constraint.

**Results:**

To favor the property of the hierarchical interaction constraint, we proposed to create groups composed of the biomarker main effect and its interaction with treatment and to perform the bi-level selection on these groups. We proposed two weighting approaches (Single Wald (SW) and likelihood ratio test (LRT)) for the adaptive lasso method. The selection performance of these two approaches is compared to alternative lasso extensions (adaptive lasso with ridge-based weights, composite Minimax Concave Penalty, group exponential lasso and Sparse Group Lasso) through a simulation study. A RCT (NSABP B-31) randomizing 1574 patients (431 events) with early breast cancer aiming to evaluate the effect of adjuvant trastuzumab on distant-recurrence free survival with expression data from 462 genes measured in the tumour will serve for illustration. The simulation study illustrates that the adaptive lasso LRT and SW, and the group exponential lasso favored the hierarchical interaction constraint. Overall, in the alternative scenarios, they had the best balance of false discovery and false negative rates for the main effects of the selected interactions. For NSABP B-31, 12 gene-treatment interactions were identified more than 20% by the different methods. Among them, the adaptive lasso (SW) approach offered the best trade-off between a high number of selected gene-treatment interactions and a high proportion of selection of both the gene-treatment interaction and its main effect.

**Conclusions:**

Adaptive lasso with Single Wald and likelihood ratio test weighting and the group exponential lasso approaches outperformed their competitors in favoring the hierarchical constraint of the biomarker-treatment interaction. However, the performance of the methods tends to decrease in the presence of prognostic biomarkers.

**Supplementary Information:**

The online version contains supplementary material available at 10.1186/s12859-023-05162-x.

## Introduction

The exploration of treatment effect heterogeneity is commonly investigated in randomized clinical trials (RCT) whatever the presence or absence of overall treatment effect. A treatment may benefit only a subgroup of patients with specific clinical or biological characteristics while it has no benefit or has a harmful effect in other subgroups. This heterogeneity corresponds to the existence of statistical interactions between the treatment and patient characteristics (or treatment effect modifiers in epidemiology). The discovery of interactions has increasing relevance in the setting of precision medicine [1] since these interactions allow to identify biomarkers or to develop biomarker-based scores or gene signatures that distinguish patients who will benefit most from a treatment. For example, the hormone receptors for estrogen $(ER+)$ and progesterone $(PR+)$ are the first biomarkers used as predictive factors of response to hormone therapy in breast cancer patients. The expression of at least one of the two receptors allows the selection of patients who will benefit from tamoxifen or other hormonotherapy [2, 3]. It is therefore important to identify biomarker-treatment interactions in RCTs [4, 5] since this is a key point for improving precision medicine [1].

In general, the identification of biomarker-treatment interactions through a limited number of biomarkers in RCTs starts firstly by developing a multivariable regression model that includes both the treatment indicator and selected biomarkers (main effects), and then by testing biomarker-treatment interactions. This follows the well-established condition used by statisticians, called the hierarchical constraint of interaction, that an interaction may be in a model if the corresponding main effects are also in the model [6–8]. The interest to encourage the development of a hierarchical model is multiple. The first reason is interpretability. It is difficult to interpret a model including an interaction without the corresponding main effects. The second reason is to respect the notion of practical sparsity defined by Bien et al. [8] by decreasing the number of measured variables in a model, which is particularly interesting in modeling for prediction. The third reason is that according to Cox [6] focusing on large main effects can lead to more appreciable interactions. In this paper, we focus on the treatment effect (which is forced in the model) associated to a survival outcome and the regression model will be the Cox proportional hazard (PH) regression model [9]. Different strategies can be used for the selection of biomarker-treatment interactions: all or a list of biologically plausible biomarker-treatment interactions are tested among the selected biomarkers (i) by adding one at a time to the main effects model or (ii) by adding all together. However, in the rare case of qualitative interaction i.e an opposite biomarker effect in the two arms, this strategy may potentially miss true interactions. In high-dimensional data characterized by a large number of biomarkers compared to the sample size ($p \gg n$), the selection of biomarkers is currently performed using the lasso penalization (Least Absolute Shrinkage and Selection Operator) [10] and/or its different extensions [11–14]. When the objective is to evaluate biomarker-treatment interactions, a previously chosen 2-step approach of identifying the biomarker-treatment interactions after penalizing all main effects did not perform satisfactorily in [15] and therefore was not included in the current paper. A naive selection procedure would consist in using the penalized approach including all the biomarkers and their interactions with treatment (2*p* dimension). In fact, it is difficult to establish a list of biomarker-treatment interactions of interest to be tested regarding the high number of biomarkers. This naive approach may result in the selection of biomarker-treatment interactions and not necessarily their biomarkers and so to the violation of the hierarchical interaction constraint. Thus, some conditions are needed in order to favor the hierarchical constraint of interaction aiming at selecting the biomarker main effect when its interaction with treatment is selected. Bien et al. [8] proposed a modification of the standard lasso method to estimate a sparse interaction model which respects the hierarchical pairwise interaction constraint between two covariates, but their method was developed for a linear regression model. In a very recent work, Du et al. [16] proposed in a context of RCT a lasso approach for survival models to identify predictors of treatment response while forcing the hierarchical structure of interactions. An interaction between a variable and treatment is selected only when either the variable or both the variable and treatment have non-zero effects.

The objective of this paper is to develop an adaptive lasso-based approach to favor the hierarchical constraint of the biomarker-treatment interaction. In other words, to favor the selection of the biomarker main effect when its interaction with treatment is selected. Our approach differs from [16] since we seek to favor (without forcing) this hierarchical constraint. We aimed to find a compromise for high-dimensional data between the need for sparse model selection and the need for the hierarchical constraint.

The paper is organized as follows. In section 2, we present the full Cox PH model with biomarker and biomarker-treatment interaction in high-dimensional data and we describe the proposed approaches and possible alternative methods adapted to our research question. In section 3, we conduct a simulation study to evaluate the selection performance of the different methods across null and several alternative scenarios. In section 4, we illustrate the different approaches through the NSABP B-31 randomized trial aiming to evaluate the effect of adjuvant trastuzumab on distant-recurrence free survival in early breast cancer patients.

## Methods

Assume a RCT which randomizes *n* patients into an experimental arm and a control arm. The main objective is to evaluate the treatment effect on a survival outcome estimated by a Cox PH model [9]. Let *T* be the treatment indicator, which takes two values: + 1/2 for the experimental arm and − 1/2 for the control arm. Assume *X* a ($n \times p$) matrix of *p* standardized biomarkers, where *n* is the sample size. For simplicity, no clinical variables were considered in the regression model. The full Cox PH model including *p* biomarkers and their interaction with treatment is defined as follows:

1 $$\begin{aligned} \lambda (t \mid T, X)=\lambda _{0}(t) \exp \left( \alpha T + \sum _{j=1}^{p} \beta _{j} X_{j}+\sum _{j=1}^{p} \gamma _{j} X_{j} T\right) , \end{aligned}$$

where $\lambda _{0}(t)$ represents the baseline hazard function, $\alpha$, $\beta = \left( \beta _{1}, \ldots , \beta _{p}\right) ^T$ represent the main effects (i.e the log Hazard Ratio noted log HR) and $\gamma = \left( \gamma _{1}, \ldots , \gamma _{p}\right) ^T$ the regression coefficients associated to the biomarker-treatment interactions. The parameters of model 1 are estimated by maximising the penalized partial log-likelihood, $\ell _p$, under the lasso [10] constraints:

2 $$\begin{aligned} \displaystyle \ell _{p}(\alpha , \beta , \gamma \mid T, X) = \ell (\alpha , \beta , \gamma \mid T, X) \, -\lambda \left( \sum _{j=1}^{p}\left| \beta _{j}\right| + \sum _{j=1}^{p}\left| \gamma _{j}\right| \right) \end{aligned}$$

where $\ell$ is the partial log-likelihood function of model 1, and $\lambda$ is the regularization parameter calculated by *k*-fold cross-validation $(\lambda \ge 0)$.

As this optimization process does not set any constraint on the selection of interactions and their corresponding main effects, it does not favor the hierarchical constraint of biomarker-treatment interaction. To respect the hierarchical interaction constraint (also known as “heredity” or being “hierarchically well-formulated” [17–19]) in model 1, the following mathematical constraint should be respected:

$$\begin{aligned} \widehat{\gamma }_{j} \ne 0 \quad \Longrightarrow \quad \hat{\beta }_{j} \ne 0, \quad j = 1, \dots , p. \end{aligned}$$

Since treatment is included in the model, this constraint corresponds to a strong hierarchy [8] i.e when a biomarker-treatment interaction effect is nonzero then the corresponding main effects (treatment and biomarker) should be in the model.

To favor the property of the hierarchical interaction constraint, we propose to express this constraint as a bi-level selection problem. In the first step, we create *p* groups composed by the biomarker main effect and by the associated biomarker-treatment interaction. In the second step, we perform the bi-level selection (both of the groups and within the groups). The reparametrization of the model 1 for a bi-level selection may then be written as follows:

3 $$\begin{aligned} \lambda (t \mid T, X)=\lambda _{0}(t) \exp \left( \alpha T + \sum _{j=1}^{p}\left( \theta _{j} V_{j}+\theta _{p+j} V_{p+j}\right) \right) , \end{aligned}$$

where $V_j = X_j$ and $V_{p+j} = X_{j} T$ define the 2 elements of group *j*, $j = 1, \dots , p$. $\varvec{V}$ is a matrix of $(n \times 2p)$ elements which can be noted as follows:

$$\begin{aligned} \varvec{V}=(\varvec{X} \mid \varvec{X} T). \end{aligned}$$

The first *p* columns correspond to the columns of the biomarker matrix $\varvec{X}$ and the columns from $p+1$ to 2*p* represent the pairwise product of $\varvec{X}$ and *T*. $\theta =(\theta _1,\dots ,\theta _p,\theta _{p+1},\dots ,\theta _{2p})^T$ represents the vector of regression coefficients (the first *p* coefficients are associated to the biomarkers and the remainder are associated to the biomarker-treatment interactions).

Based on this new parametrization, we present our bi-level selection procedure based on the adaptive lasso and other alternative lasso extensions.

### Bi-level selection procedure based on the adaptive lasso

We extended the adaptive lasso penalized regression [11, 20] for a bi-level selection by constructing a penalty function including both the coefficients associated with the biomarkers and their interactions with treatment. The choice to use this method is motivated by the fact that the sizes of the main effect of a biomarker and its interaction with the treatment can be very different, and the adaptive lasso method may assign adaptive weights to each coefficient in order to penalize them differently. The adaptive lasso penalized regression consists in maximizing the penalized partial log-likelihood function

4 $$\begin{aligned} \displaystyle \ell _{p}(\alpha , \theta \mid T, X) = \ell (\alpha , \theta \mid T, X) \, -\lambda \sum _{j=1}^{p}\left( \omega _{j} \left| \theta _{j}\right| + \omega _{p+j} \left| \theta _{p+j}\right| \right) , \end{aligned}$$

where $\ell$ is the partial log-likelihood function of model 3 and $\lambda$ is the regularization parameter. For each group *j*, $\left( \omega _{j}, \omega _{p+j}\right)$ represent the adaptive weights assigned to the coefficients associated with the biomarker main effect and its interaction with treatment, respectively. Contrary to the Eq. 2 that assigns an identical penalty to all biomarkers and their interaction with treatment, Eq. 4 assigns a specific penalty for each biomarker and its interaction with treatment contributing also to encourage the hierarchical constraint.

The adaptive weights are estimated in a preliminary stage. We propose two types of weights based on statistical tests as described below.

#### Single wald (SW)

Single Wald (SW) weighting is inspired by our previous work [21], where we showed that weighting strategies based on the Wald statistic gives good results for biomarker selection in the case of biomarkers grouped by pathways. The SW weighting strategy assigns the same weight to the biomarker and its interaction with treatment. This weight is equal to the inverse of the Wald statistic ($W_{j}$) which is defined by a univariable Cox PH model incorporating only the biomarker-treatment interaction (i.e. $\lambda (t \mid T, X)=\lambda _{0}(t) \exp (\gamma _{j} X_{j} T)$).

For each group *j*, the specific adaptive weight is also set as:

5 $$\begin{aligned} \left( \omega _{j}, \omega _{p+j}\right) = \left( \frac{1}{W_{j}}, \frac{1}{W_{j}}\right) . \end{aligned}$$

A strong statistical association between biomarker-treatment interaction and survival outcome (high $W_{j}$) corresponds to a small weight and thus a smaller penalty in model 4. As we give the same weight to the interaction and the main effect, this allows to favor the selection of the latter when the former is selected.

#### Likelihood ratio test (LRT)

The likelihood ratio test (LRT) is used to evaluate the statistical significance of a vector of coefficients. In particular, it can be used to evaluate the relative goodness of fit of two nested regression models. To estimate the adaptive weights, we considered the following nested models:

- The basic model with only the treatment indicator
  $$\begin{aligned} M_{0} :\lambda \left( {t,\alpha \left| T \right.} \right) = \lambda _{0} (t)\exp \left( {\alpha T} \right) \end{aligned}$$
- The main effects model
  $$\begin{aligned} M_{1} : \lambda (t, \alpha , \beta \mid T, X)=\lambda _{0}(t) \exp \left( \alpha T + \beta _j X_j \right) , \quad j = 1,\ldots , p \end{aligned}$$
- The main effects + biomarker-treatment interaction model
  $$\begin{aligned} M_{2} :\lambda \left( {t,\alpha ,\beta ,\gamma \left| T \right.,X} \right) = \lambda _{0} \left( t \right)\exp \left( {\alpha T + \beta _{j} X_{j} + \gamma _{j} X_{j} T} \right),j = 1, \ldots ,p \end{aligned}$$

Based on these three models, we consider the following hypotheses:

- For models $M_{0}$ *versus* $M_{2}$
  $$\begin{aligned} H_{0} &:\beta _{j} = \gamma _{j} = 0\\ H_{1} &:\left\{ {\beta _{j} \;{\text{and}}/{\text{or}}\;\gamma _{j} } \right\} \ne 0 \end{aligned}$$
- For models $M_{1}$ *versus* $M_{2}$
  $$\begin{aligned} &H_{0} : \gamma _j = 0 \\&H_{1} : \gamma _j \ne 0 \end{aligned}$$

For each biomarker, we consider the partial likelihood ratio test statistics between models $M_{0}$ and $M_{2}$ ($\Lambda _{M_{2}/M_{0}}$) and between $M_{1}$ and $M_{2}$ ($\Lambda _{M_{2}/M_{1}}$). For each group *j*, $j = 1, \ldots , p$, the adaptive weights assigned to the biomarker main effect $\omega _{j}$ and the biomarker-treatment interaction $\omega _{p+j}$ were defined

6 $$\begin{aligned} \left( \omega _{j}, \omega _{p+j}\right) = \left( \frac{1}{\Lambda _{M_{2}/M_{0}}^{(j)}}, \frac{1}{\Lambda _{M_{2}/M_{1}}^{(j)}}\right) . \end{aligned}$$

It is important to note that we assigned to a biomarker a weight based on the likelihood ratio test statistic ($\Lambda _{M_{2}/M_{0}}^{(j)}$) comparing the basic model including treatment indicator only and the model with treatment indicator, biomarker and its interaction with treatment in order to underpenalize the biomarker in presence of evidence of interaction with treatment. Using the likelihood ratio test statistic ($\Lambda _{M_{1}/M_{0}}^{(j)}$) between the basic model and model with treatment and biomarker would not have allowed to take advantage of the presence of interaction in the weight assigned to the biomarker and thus would not have encouraged the hierarchical constraint selection. With this weighting strategy, if the value of $\Lambda _{M_{2}/M_{0}}$ is high, the biomarker likely has a prognostic and/or predictive role, and thus the weight given to a coefficient $\beta _j$ of the biomarker $X_j$ ($\omega _{j} = 1/\Lambda _{M_{2}/M_{0}}^{(j)}$) is small, which increases the chances that it will be selected in the final model. For a predictive biomarker *j*, the coefficient of its interaction with treatment is considered non-zero ($\gamma _j \ne 0$), therefore the values of the likelihood ratio tests ($\Lambda _{M_{2}/M_{0}}$ and $\Lambda _{M_{2}/M_{1}}$) are far from zero and then the weights given to the biomarker main effect and the biomarker-treatment interaction ($\omega _{j}$ and $\omega _{p+j}$) are low. This favors the selection of the biomarker-treatment interaction and its biomarker main effect. On the other hand, if a biomarker *j* is a prognostic biomarker, i.e. has a large main effect but does not interact with treatment ($\beta _j \ne 0$ and $\gamma _j = 0$) then the value of $\Lambda _{M_{2}/M_{0}}$ is high but $\Lambda _{M_{2}/M_{1}}$ is low. Therefore, the value of weight $\omega _{j}$ is low and weight $\omega _{p+j}$ is high, this encourages selection of the biomarker main effect rather than selection of its interaction with treatment.

### Alternative approaches

We present several extensions of the standard lasso that have been developed to perform group and within-group selection. They differ according to the penalized function used. We have adapted them in the context of a bi-level selection of biomarker main effects and biomarker-treatment interactions and compared them to our proposed approach. These methods include the adaptive lasso with ridge penalty-based weights, the composite Minimax Concave Penalty (cMCP), the group exponential lasso (gel) and the Sparse Group Lasso (SGL) and are presented below.

#### Adaptive lasso with a ridge-based weights

In the context of the identification of predictive biomarker in RCT, Ternès et al. [15] compared several approaches for biomarker-treatment interaction selection in high-dimensional Cox regression models. Among these approaches, they studied different extensions of the standard lasso method that penalize both the biomarker main effects and the biomarker-treatment interactions but without addressing the question of the hierarchy constraint. The authors showed that the adaptive lasso method with weights estimated by the ridge method (presented below) works reasonably well to select biomarker-treatment interactions. The ridge regression [22] is a penalization method often used in the case of strong correlation between variables. This method introduces a penalty term to the partial log-likelihood function of the model 3 to limit the instability of the coefficients. The ridge penalty term corresponds to the $L_2$ norm of the regression coefficients and the penalized partial log-likelihood function is defined as follows:

7 $$\begin{aligned} \displaystyle \ell _{p}(\alpha , \theta \mid T, X)=\ell (\alpha , \theta \mid T, X)-\lambda \sum _{j=1}^{p}\left( \theta _{j}^{2}+\theta _{p+j}^{2}\right) . \end{aligned}$$

By increasing the value of the regularization parameter $\lambda$, the values of the regression coefficients shrunk towards zero. Unlike the lasso method, the ridge method does not set any regression coefficient to 0. Therefore, it does not perform variable selection. On the other hand, the ridge method provides a robust estimation of the parameters by reducing the variance. It also gives good predictive performances in terms of bias-variance trade-off [23]. Maximization of the penalized partial log-likelihood function 7 estimates ridge regression coefficients $\tilde{\theta }_{j}^{R}$ and $\tilde{\theta }_{p+j}^{R}$ for biomarker main effect and biomarker-treatment interaction, respectively. The adaptive weights given to the biomarker main effect and its biomarker-treatment interaction are defined as the inverse of the absolute value of these regression coefficients,

8 $$\begin{aligned} \left( \omega _{j}, \omega _{p+j}\right) = \left( \frac{1}{\left| \tilde{\theta }_{j}^{R}\right| }, \frac{1}{\left| \tilde{\theta }_{p+j}^{R}\right| }\right) . \end{aligned}$$

#### Composite minimax concave penalty (cMCP)

The composite Minimax Concave Penalty (cMCP) method [24, 25] performs bi-level selection by combining individual and group variable penalties. It allows to select the influent groups and the influent variables of these groups. Considering the model 3 the penalized log-likelihood function of cMCP is written

9 $$\begin{aligned} \displaystyle \ell _{p}(\alpha , \theta \mid T, X) = \ell (\alpha , \theta \mid T, X) - \sum _{j=1}^{p} f_{O}\left( f_{I}\left( \theta _{j}\right) + f_{I}\left( \theta _{p+j}\right) \right) , \end{aligned}$$

where $f_{O} = f_{\lambda , b}$ and $f_{I} = f_{\lambda , a}$ are the outer and inner MCP functions [26], respectively (a> 0 and b > 0 are the shape parameters for the outer and inner penalties, respectively). The MCP is defined on the support $[0, \infty )$ as

10 $$\begin{aligned} f_{\lambda , a}(\theta )=\left\{ \begin{array}{ll}\lambda \theta -\frac{\theta ^{2}}{2 a}, &{} \text{ if } \theta \le a \lambda \\ \frac{1}{2} a \lambda ^{2}, &{} \text{ if } \theta > a \lambda \end{array}\right. , \text{ with } \lambda \ge 0. \end{aligned}$$

#### Group exponential lasso (gel)

Like the cMCP method, the group exponential lasso (gel) [27] method also performs bi-level variable selection. It maximizes the penalized partial log-likelihood function similar to cMCP, with an outer penalty equal to the exponential penalty and an inner penalty equal to the standard lasso penalty. The exponential penalty is defined on the support $[0, \infty )$ as

11 $$\begin{aligned} f_{\lambda , \tau }(\theta )=\frac{\lambda ^{2}}{\tau }\left\{ 1-\exp \left( -\frac{\tau \theta }{\lambda }\right) \right\} , \end{aligned}$$

where $\tau$ is the rate of exponential decay. When $\tau \longrightarrow 0$, the gel method selects a model equivalent to the standard lasso, while when $\tau \longrightarrow 1$, it selects a model equivalent to the group lasso [13].

#### Sparse group lasso (SGL)

Simon et al. [12] proposed the Sparse Group Lasso (SGL) method to promote sparsity at two different levels: “sparsity by group” and “sparsity within group” by performing the selection of relevant groups and within groups. The penalized partial log-likelihood function of the SGL method is

12 $$\begin{aligned} \displaystyle \ell _{p}(\alpha ^*, \theta \mid T, X)&= \ell (\alpha ^*, \theta \mid T, X) \, - (1-\alpha ^*)\lambda \sum _{j= 1}^{p}\sqrt{2}\left\| \theta ^{(j)}\right\| _{2} - \alpha ^*\lambda \left\| \theta \right\| _{1} \\&=\ell (\alpha ^*, \theta \mid T, X) \, - (1-\alpha ^*)\lambda \sum _{j= 1}^{p}\sqrt{2 \left( \theta _{j}^2+\theta _{p+j}^2\right) } - \alpha ^*\lambda \sum _{j= 1}^{p} \left( \left| \theta _{j}\right| +\left| \theta _{p+j} \right| \right) ,\\ \end{aligned}$$

where $\alpha ^* \in [0,1]$. If $\alpha ^*=1$ or $\alpha ^*=0$, the SGL penalty is equal to the standard lasso and group lasso, respectively. Note the group lasso method itself was not included in our study because it does not perform individual biomarker selection within the predefined groups but only group selection. This does not address our goal of sparse model selection, as this approach may falsely select the biomarker-treatment interaction when its main effect is selected. As the SGL method does not adjust for clinical variables, treatment was considered as a penalized variable in a separate group. In contrast, the other methods presented above allow to include the treatment in the final model without penalizing it. In fact, with the adaptive lasso method we assign a zero weight to the treatment coefficient so that it is not penalized and included in the final model. The cMCP and gel methods assign to the group “0” the coefficients to be included in the model without being penalized.

For all these approaches described above as for the standard lasso, the regression coefficients are estimated in 2 steps. Firstly, the optimal penalty term $\hat{\lambda }_{cvl}$ is selected by *k*-fold cross-validation (*k* set to 5) in maximizing the cross-validated log-likelihood [28, 29] Secondly, the penalized log-likelihood function is maximized.

## Simulation study

We conducted a simulation study to compare the selection performance of methods described so far in the context of high-dimensional data for a survival outcome and in particular to investigate the ability of the methods to favor hierarchical constraint of biomarker-treatment interactions, i.e., to identify relevant interactions with their corresponding biomarker main effects.

### Data simulation

We generated $n=3000$ patients drawing a RCT divided into a training and a validation set with equal sample size ($n=1500$) and $p = 500$ biomarkers (gene expressions) leading to 500 biomarker-treatment interactions. Patients were randomly assigned to each treatment arm with probability of 0.5 (ratio 1:1). The treatment was coded + 0.5 for the experimental arm and − 0.5 for the control arm. The biomarkers were generated from a standard multivariate Gaussian distribution (means $\mu _{1}=\cdots =\mu _{p}=0$ and standard deviations $\sigma _{1}=\cdots =\sigma _{p}=1$). The biomarker correlation structure was defined as autoregressive by 20-biomarker blocks; in each block the correlation between two biomarkers *i* and *j* was set to $\rho _{i j}=0.7^{\left| i-j\right| }$. We generated survival times using an exponential distribution with a median survival of 1 year. Censoring times were generated from a uniform distribution *U*(2, 5), reflecting a trial with a 3-year accrual period and a 2-year follow-up.

### Simulated scenarios

Different scenarios of RCT were generated by varying $q_{Po}$, the number of biomarkers associated to the survival outcome (prognostic biomarkers) and $q_{Pe}$, the number of biomarkers which interact with the treatment (predictive biomarkers) (Table 1). We first considered a null scenario with neither prognostic nor predictive effect for any biomarker (scenario 1); then two alternative scenarios 2 and 3 with no prognostic biomarker and at least one biomarker that interacts with the treatment; finally, the alternative scenario 4 with both prognostic and predictive biomarkers. For this last scenario, the 10 prognostic biomarkers were chosen to be necessarily different from the 10 predictive biomarkers and they may be considered as noise in detection of the true interactions. We extended this last scenario by increasing the number of biomarkers from $p = 500$ to $p = 5000$ and keeping 10 predictive biomarkers and 10 prognostic biomarkers (scenario 4b). The treatment effect was generated with an effect size of $\alpha =ln(HR)=ln(0.5)$. Prognostic and predictive biomarkers were generated with an effect size of $\beta = \gamma = ln (0.5)$. For each scenario, 500 replications were simulated.

**Table 1:** Scenarios of the simulation study

Scenarios		Biomarker effect: $ln(HR)=\beta \pm 0.5\gamma$		Average censoring probability
		*Ctrl:* $\beta -0.5\gamma$	*Exp:* $\beta +0.5\gamma$	
1	No effect	0	0	0.10
2	$q_{Pe}= 1$ predictive biomarker	-$0.5 \, ln(0.5)$	$0.5 \, ln(0.5)$	0.13
3	$q_{Pe}= 10$ predictive biomarkers	-$0.5 \, ln(0.5)$	$0.5 \, ln(0.5)$	0.20
4	$q_{Pe}= 10$ predictive biomarkers	-$0.5 \, ln(0.5)$	$0.5 \, ln(0.5)$	0.31
	$q_{Po} = 10$ prognostic biomarkers	*ln*(0.5)	*ln*(0.5)	

*HR* hazard ratio, *Ctrl* control arm, *Exp* experimental arm, $q_{Po}$ number of prognostic biomarkers, $q_{Pe}$ number of predictive biomarkers

### Evaluation criteria

For evaluating the ability of the different methods to select (i) true biomarker-treatment interactions (predictive biomarkers) and (ii) their corresponding main effects, i.e., to respect the hierarchical constraint of biomarker-treatment interactions, different criteria of selection performance were used. For each simulated data set, we first report the number of selected biomarker-treatment interactions $(n_{Pe})$ and the number of selected main effects corresponding to the selected interactions $(n_{Po})$. Second, the false discovery rate (FDR, proportion of selected parameters that are noninfluent) [30] and false negative rate (FNR, proportion of influent parameters that are not selected) [31] were calculated for the biomarker-treatment interaction and the corresponding main effect parameters, separately:

$$\begin{aligned} FDR = FP/(TP+FP), \; FNR = FN/(TP+FN) \end{aligned}$$

with i) TP the number of influent parameters that were selected, ii) FP the number of noninfluent parameters that were selected and iii) FN the number of influent parameters that were not selected. Since our objective is to favor the hierarchical constraint, the calculation of the FDR and FNR of main effects is based only on the main effects of the selected interactions. A biomarker main effect is considered as a true positive or false positive if its interaction with treatment is a true positive or false positive, respectively. In addition, a biomarker main effect is considered as a false negative if its interaction with treatment is false negative.

We also investigated the impact of favoring the hierarchical constraint of biomarker-treatment interaction by measuring the difference in concordance indices of a score defined as the product between the coefficients of the interactions retained in the training set and their biomarkers [15, 32] and the survival time, between the 2 treatment arms. This approach was originally proposed by Schemper [33]. We estimated these concordances via the C-statistic of Uno [34] and we calculated the difference of this statistic between the two arms ($\Delta$C-statistic) both in the training and validation sets, respectively. A high value of the difference indicates a high interaction strength. In addition, the usual Uno C-statistic estimated from the full linear predictor of the selected models was reported.

We used the simdata function of the biospear [35, 36] R package for the simulation study and the following packages for the statistical analyses: glmnet [37, 38] for the adaptive lasso (LRT) and (SW) methods; biospear [35] for the adaptive lasso (ridge) method; grpreg [39] for the cMCP (default values for the tuning parameters a and b were considered) and gel methods with $\tau = 1/3$ [27] and SGL [40] for the SGL method with the default value of the $\alpha$ parameter equal to 0.95. We used the default grid values in the R packages to select the tuning parameter $\lambda$.

### Results

**Fig. 1:**
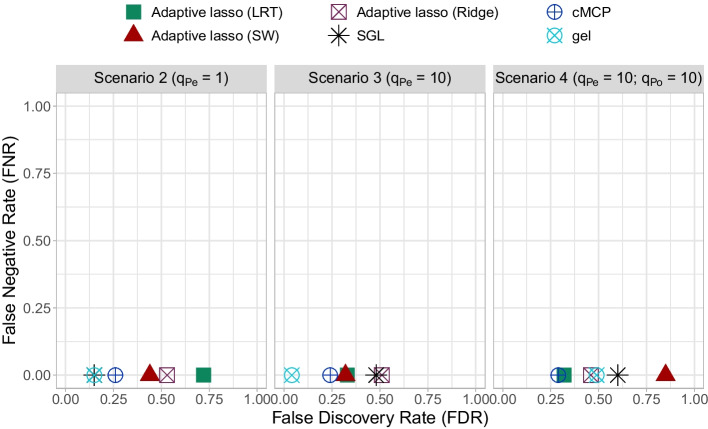
False negative rate (FNR) versus false discovery rate (FDR) of biomarker-treatment interactions in alternative scenarios

**Fig. 2:**
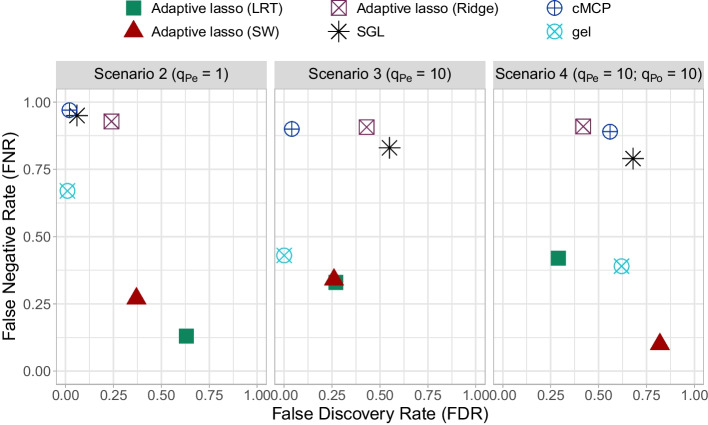
False negative rate (FNR) versus false discovery rate (FDR) of biomarker main effects corresponding to selected interactions in alternative scenarios

Table 2 details the selection performance of biomarker-treatment interactions and of their corresponding main effects for the null and the three alternative scenarios. For a given scenario, the first and second rows correspond to the selection performance of biomarker-treatment interactions and of the corresponding main effects. Within each row, the average number of selected biomarkers ($n_{Pe}$ or $n_{Po}$), its 2 components (TP and FP) and the average FDR and FNR are reported for the 6 methods. In addition, Figs. 1 and 2 complete this table by allowing to directly identify the methods offering the best trade-off between FDR and FNR (for biomarker-treatment interactions and their corresponding main effects, respectively) across the different scenarios.

In the null scenario 1, with no signal ($q_{Pe} = 0$ predictive biomarker and $q_{Po} = 0$ prognostic biomarker), the adaptive lasso method with LRT and SW weights selected more false interactions and corresponding main effects than the other methods. For example, the adaptive lasso (LRT) method selected on average $n_{Po} = 3.96$ main effects corresponding to $n_{Pe} = 9.96$ selected interactions. This translates into high FDR ($>80\%$) for interactions and main effects. In contrast, the cMCP and gel methods selected only $n_{Po} = 0.01$ main effects on average, corresponding to $n_{Pe} = 0.68$ and 0.44 selected interactions, respectively. The SGL method did not select any interactions.

In the alternative scenario 2 ($q_{Pe} = 1$ predictive biomarker and $q_{Po} = 0$ prognostic biomarker), all methods identified the true biomarker-treatment interaction (TP=1). The adaptive lasso method with LRT and SW weights selected on average $n_{Po} = 4.79$ and 3.67 main effects, corresponding to $n_{Pe} = 8.89$ and 6.71 selected interactions ($n_{Po}/n_{Pe} \ge 54\%$), respectively. This yields to FDR/FNR values for interactions of 0.72/0.00 and 0.44/0.00, respectively, and for the main effects of 0.63/0.13 and 0.37/0.27, respectively. The other methods selected fewer interactions (varying from 1.37 to 4.31 in average) and corresponding main effects (less than 1 on average). For example, the SGL method had an average number of selected interactions of $n_{Pe} = 1.37$ and corresponding selected main effects of $n_{Po} = 0.11$. Similarly, the cMCP method selected only $n_{Po} = 0.05$ main effects corresponding to $n_{Pe} = 2.28$ selected interactions. Besides, the SGL and cMCP and gel methods have the lowest FDR (Figs. 1 and 2).

In the alternative scenario 3 ($q_{Pe} = 10$ predictive biomarkers and $q_{Po} = 0$ prognostic biomarker), all methods selected the 10 true biomarker-treatment interactions (TP = 10). The adaptive lasso (LRT and SW) methods had similar selection performance. Both favored the hierarchical biomarker-treatment interaction constraint ($n_{Po}/n_{Pe} \ge 60\%$) more than the other methods. Indeed, they selected $n_{Pe} = 15.51$ and 15.18 predictive biomarkers and $q_{Po}=9.54$ and 9.15 corresponding main effects, respectively. The gel method gives similar results with $n_{Pe} =10.48$ interactions and $n_{Po} =5.73$ corresponding main effects. In terms of interactions, the proposed approaches had an FDR around 0.32 higher than that of the gel method (0.04). In terms of main effects, the adaptive lasso (LRT and SW) had a lowest FDR/FNR balance of main effects equal to 0.27/0.33 and 0.26/0.34, respectively. On the other hand, the gel method had a lower FDR equal to 0 and a higher FNR equal to 0.43.

In the alternative scenario 4 ($q_{Pe} = 10$ predictive biomarkers and $q_{Po}=10$ prognostic biomarkers) and as in the scenarios 2 and 3, all methods selected the 10 true biomarker-treatment interactions. The adaptive lasso (LRT and SW) and gel methods favored the hierarchical biomarker-treatment interaction constraint more than the other methods ($n_{Po}/n_{Pe} \ge 56\%$). However, the adaptive lasso (SW) method selected more false positive biomarkers than the others: 60.89 noninfluent among 70.88 selected interaction compared to 5.18/15.15 and 9.69/19.67 for the adaptive lasso (LRT) and gel methods, respectively. It had also the highest FDR and the lowest FNR: the FDR/FNR balance of the interaction was equal to 0.85/0.00, and that of the corresponding main effects was equal to 0.82/0.10. The adaptive lasso (LRT) outperformed the other competitive approaches by the best FDR/FNR balance for interactions (0.32/0.00), and for corresponding main effects (0.29/0.42).

When comparing the selection performance of the different methods in increasing the number of true predictive biomarkers from 1 (scenario 3) to 10 (scenario 4), one important result is the behaviour of the adaptive lasso (SW) with respect to the adaptive lasso (LRT). The performance of the former decreases while that of the latter is comparable in terms of FDR of both interactions and corresponding main effects (Figs. 1 and 2).

Overall, the $\Delta$C-statistics estimated on the training set and for the different methods are similar within a scenario since all methods selected the true biomarker-treatment interactions (Additional file 1: Fig. S1A) and the coefficients of the selected false interactions are close to zero (data not shown). On average $\Delta$C-statistics were around 0.2 (scenario 2), 0.5 (scenario 3) and 0.27 (scenario 4) and it decreases very slightly on the validation set. For this scenario 4, we observed that the adaptive lasso (SW) method had an estimated $\Delta$C-statistics in the training set higher than 0.3 due to a higher number of selected false interactions than the other methods (overfitting). The difference in scenario 3 compared to the scenario 2 is explained by the higher number of true biomarker-treatment interactions selected. The reason of a decreasing $\Delta$C-statistics in scenario 4 compared to scenario 3 is due to lower regression coefficients of selected interactions (lower interaction strength). The predictive performance of the selected models, evaluated by the C-statistic on the training set (Additional file 1: Fig. S1B), is globally similar across the different approaches within a scenario: around 0.65 for scenario 2, 0.75 for scenario 3 and 0.85 for scenario 4 with a large variability for the adaptive lasso (SW). It slightly decreases on the validation set with a distribution of C-statistic quasi-identical between the approaches for a given scenario except for the adaptive lasso (SW) in scenario 4 for which the decrease is more marked. The property of favoring the hierarchical interaction constraint for the adaptive Lasso (SW and LRT) did not translate into higher C-statistic values.

When increasing the number of biomarkers in scenario 4 (*p* from 500 to 5000 biomarkers), called scenario 4b, the main differences are (1) the gel approach favored mostly the hierarchical biomarker-treatment interaction constraint with $n_{Po}/n_{Pe} > 70\%$ followed by the adaptive lasso (LRT and SW) with $n_{Po}/n_{Pe} < 50\%$ (Additional file 1: Table S1), and (2) the adaptive lasso (SW) selected a higher number of false interactions. In terms of $\Delta$C-statistics evaluating the strength of interaction, we observe similar results as in scenario 4 (with *p*=500) but with a larger difference between the training and validation for the adaptive lasso (SW) (Additional file 1: Fig. S2A). Only the adaptive lasso (ridge) reports a small value for this $\Delta$C-statistic since it selects the smallest number of interactions among all approaches (Additional file 1: Table S1). The c-statistics estimated on the training set (Additional file 1: Fig. S2B) are similar across the different approaches except for the adaptive lasso (ridge) yielding a smaller c-statistic with a large variability. It is explained by the small number of true interactions selected by this approach (Additional file 1: Table S1). Although the c-index is slightly lower on the validation set as compared to the training set for the two approaches with the higher FDR i.e. the adaptive lasso (SW) and (ridge), the c-statistics are quite similar between the training and validation sets. This trend is consistent across the alternative scenarios and could be explained by the similar way the training and validation sets are generated with strong effect sizes among candidate biomarkers with a specific correlation structure (see subsection Data simulation). Compared to scenario 4 (with *p* = 500), the c-indices of scenario 4b (with *p* = 5000) are numerically smaller but still in the same range illustrating the ability of these approaches to capture the true signal (characterized by the effect size of true biomarkers and correlation patterns) and distinguish this signal from the noise (characterized by the high number of biomarkers unrelated to the outcome) in the simulations we performed.

**Table 2:** Selection performance of biomarker-treatment interactions and main effects corresponding to the selected interactions

			AL(LRT)	AL(SW)	AL(ridge)	SGL	cMCP	gel
Scenario 1 $q_{Pe} = 0$ $q_{Po} = 0$	Interactions	$n_{Pe}$	9.96	13.02	1.95	0.00	0.68	0.44
		FP	9.96	13.02	1.95	0.00	0.68	0.44
		FDR	0.95	0.97	0.95	-	0.24	0.20
	Main effects	$n_{Po}$ $(n_{Po}/n_{Pe})$	3.96 (40%)	5.68 (44%)	0.24 (12%)	0.00	0.01 (1%)	0.01 (2%)
		FP	3.96	5.68	0.24	0.00	0.01	0.01
		FDR	0.85	0.88	0.17	-	0.01	0.01
Scenario 2 $q_{Pe} = 1$ $q_{Po} = 0$	Interactions	$n_{Pe}$	8.89	6.71	4.31	1.37	2.28	1.54
		TP / FP	1.00/7.89	1.00/5.71	1.00/3.31	1.00/0.37	1.00/1.28	1.00/0.54
		FDR / FNR	0.72/0.00	0.44/0.00	0.53/0.00	0.15/0.00	0.26/0.00	0.15/0.00
	Main effects	$n_{Po}$ $(n_{Po}/n_{Pe})$	4.79 (54%)	3.67 (55%)	0.48 (11%)	0.11 (8%)	0.05 (2%)	0.34 (52%)
		TP / FP	0.87/3.93	0.73/2.94	0.07/0.41	0.05/0.06	0.03/0.02	0.33/0.01
		FDR / FNR	0.63/0.13	0.37/0.27	0.24/0.93	0.06/0.95	0.02/0.97	0.01/0.67
Scenario 3 $q_{Pe} = 10$ $q_{Po} = 0$	Interactions	$n_{Pe}$	15.51	15.18	22.40	20.20	13.68	10.48
		TP / FP	10.00/5.51	10.00/5.18	10.00/12.40	10.00/10.20	10.00/3.68	10.00/0.48
		FDR / FNR	0.33/0.00	0.32/0.00	0.51/0.00	0.48/0.00	0.24/0.00	0.04/0.00
	Main effects	$n_{Po}$ $(n_{Po}/n_{Pe})$	9.54 (61%)	9.15 (60%)	2.28 (1%)	4.09 (20%)	1.02 (7%)	5.73 (55%)
		TP / FP	6.74/2.80	6.61/2.54	0.93/1.35	1.69/2.40	0.96/0.07	5.72/0.01
		FDR / FNR	0.27/0.33	0.26/0.34	0.43/0.91	0.55/0.83	0.04/0.90	0.00/0.43
Scenario 4 $q_{Pe} = 10$ $q_{Po} = 10$	Interactions	$n_{Pe}$	15.15	70.88	19.93	26.27	14.57	19.67
		TP / FP	9.97/5.18	9.99/60.89	10.00/9.93	10.00/16.27	10.00/4.57	9.99/9.69
		FDR / FNR	0.32/0.00	0.85/0.00	0.46/0.00	0.60/0.00	0.29/0.00	0.49/0.00
	Main effects	$n_{Po}$ $(n_{Po}/n_{Pe})$	8.43 (56%)	52.40 (74%)	2.04 (10%)	6.88 (26%)	2.73 (19%)	15.56 (78%)
		TP / FP	5.80/2.63	8.99/43.41	0.91/1.13	2.07/4.82	1.11/1.62	6.05/9.51
		FDR / FNR	0.29/0.42	0.82/0.10	0.42/0.91	0.68/0.79	0.56/0.89	0.62/0.39

$q_{Pe}$ number of predictive biomarkers, $q_{Po}$ number of prognostic biomarkers, $n_{Pe}$ number of selected interactions, $n_{Po}$ number of selected main effects corresponding to the selected interactions, *FP* false positives, *TP* true positives, *FDR* false discovery rate, *FNR* false negative rate, *AL* adaptive lasso, *LRT* likelihood ratio test, *SW* single Wald

## Application

**Fig. 3:**
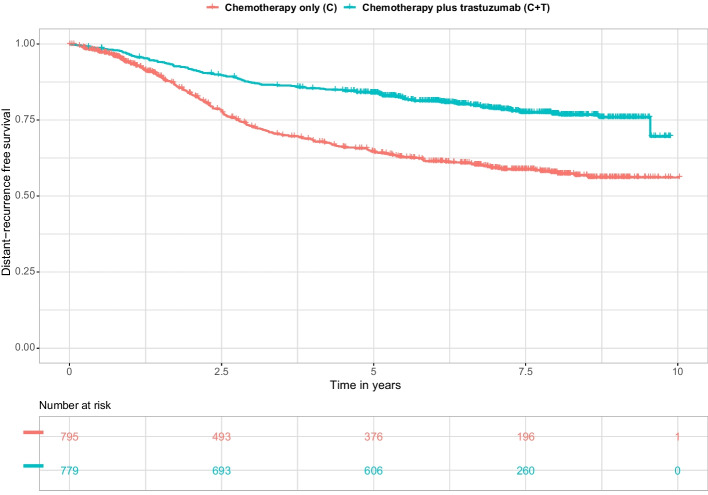
Distant-recurrence free survival curves estimated by Kaplan-Meier for the two arms of the NSABP B-31 randomized clinical trial ($$n=1574)$$

We illustrated the proposed approaches on data from a National Surgical Adjuvant Breast and Bowel Project (NSABP) B-31 randomized controlled trial evaluating the effect of adjuvant trastuzumab on the distant-recurrence free survival (DRFS) in patients with early breast cancer [41]. A total of $n = 1574$ patients were randomized into two treatment arms: chemotherapy alone (C arm, $n = 795$) and chemotherapy plus trastuzumab as adjuvant therapy (C + T arm, $n = 779$). The censoring rate was 73% (431 events for DRFS) and the 5-year DRFS was 65% [95% CI: 61% -68%] and 84% [95% CI: 81% -86%] for patients in arms C and C+T, respectively (Fig. 3). Trastuzumab in combination with chemotherapy significantly improved DRFS compared with chemotherapy alone with a *HR* = 0.46 [95% CI: 0.38 -0.56]. The proportional hazards assumption was not violated. However, this effect may not be the same across the study population, and the benefit of adding trastuzumab could varied due to the presence of gene-treatment interactions. Gene expression data had been collected for $p = 462$ genes and thus 462 potential gene-treatment interactions of potential interest.

The objective of this application is to identify gene-treatment interactions and the corresponding main effects of these genes which predict the response of trastuzumab in patients diagnosed with early breast cancer. To address the well-known problem of instability of the lasso approach on the optimal $\lambda$ and thus the selected model resulting to the fold assignment of the cross-validation process [42], we performed 500 replications by randomly dividing the dataset into a training set (60% of the original data) and a validation set (40%) each time. For evaluating the respect or not of the hierarchical interaction constraint, we calculated, on the training set, the proportion of selected models containing both the gene-treatment interaction with its corresponding main effect among the selected models containing the gene-treatment interaction (with or without its corresponding main effect). The closer the proportion is to 1, the more the hierarchical gene-treatment interaction constraint is favored in the selection procedure. We also calculated, like in the simulation study, the concordance index, $\Delta$C-statistic, between the experimental and control arms on the validation set and the overall concordance index, C-statistic, of the selected models.

**Fig. 4:**
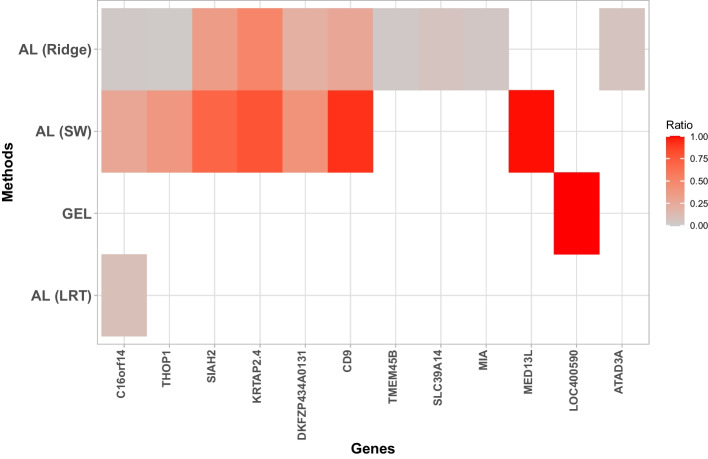
Genes for which interaction with treatment is identified, by the different methods, in at least 20% of 500 replications of the training set (representing 60% of the randomized clinical trial of breast cancer) for distant-recurrence free survival. Each square represents a gene whose the interaction with treatment is selected according to the cut-off. The intensity of the color is proportional to the percentage that a model with a gene-treatment interaction and its main effect is selected among all models selecting this gene-treatment interaction term. This allows to characterise the property of the hierarchical interaction constraint

Figure 4 shows the genes for which the interaction with treatment is selected in at least 20% of the 500 replicates (100/500) by each method. The x-axis ranks the genes most selected by the different methods down to the least selected and the y-axis ranks the method that selects the fewest gene-treatment interactions down to the one that selects the most. Each square corresponds to a gene whose interaction with treatment is selected. The intensity of the color is proportional to the percentage that the model with a gene-treatment interaction and its main effect are selected among all models selecting this gene-treatment interaction term (property of respecting the hierarchical constraint). This also characterizes the property of the hierarchical constraint. Overall, the adaptive lasso methods with ridge weighting and SW weighting identified the highest number of gene-treatment interaction with 10 and 7, respectively. Among these genes, the adaptive lasso (SW) favors the gene-treatment interaction constraint more than the adaptive lasso (ridge) method. Indeed, the proportion of model selection that includes both gene-treatment interactions with its corresponding main effects varies from 28.2% for the C16orf14 gene to 98.8% for the MED13L gene. In contrast, the highest proportion obtained with the adaptive lasso (ridge) is less than 50.6% (KRTAP2.4 gene). This means that the adaptive lasso (ridge) method selects more often the gene-treatment interaction only. The gel method selected only 1 gene (LOC400590) whose proportion of model selection with both a gene-treatment interaction and main effect is 100%. The adaptive lasso with LRT weighting method selected the C16orf14 gene respecting the property with a proportion of 9.9%. The SGL and cMCP methods selected no gene-treatment interactions more than or equal to 20% of 500 iterations.

When increasing the cut-off of selection of gene-treatment interaction model (with or without main effect) to 40 and 50%, the number of identified genes substantially decreases for all methods. Only the adaptive lasso (ridge) and (SW) methods have identified gene-treatment interactions respecting the property. Indeed, the adaptive lasso (SW) have selected 3 genes (SIAH2, KRTAP2.4, CD9) and the adaptive lasso (ridge) only the C16orf14 gene. No method has selected interactions above the cut-off of 50% (data not shown). Among the gene-treatment interactions selected, several articles [43–45] have reported that down-regulation of CD9 is associated with increased aggressiveness of breast carcinoma. Jansen et al. [46] showed that the SIAH2 protein is a predictor of disease progression in ER-positive breast cancer after tamoxifen treatment. Notably, SIAH2 messenger RNA is significantly associated with ER protein levels in primary breast tumors. In another example, Chan et al. [47] noted that SIAH2 protein levels were mostly upregulated in ER-negative breast cancer and that overexpression of SIAH2 was associated with unfavorable survival.

**Fig. 5:**
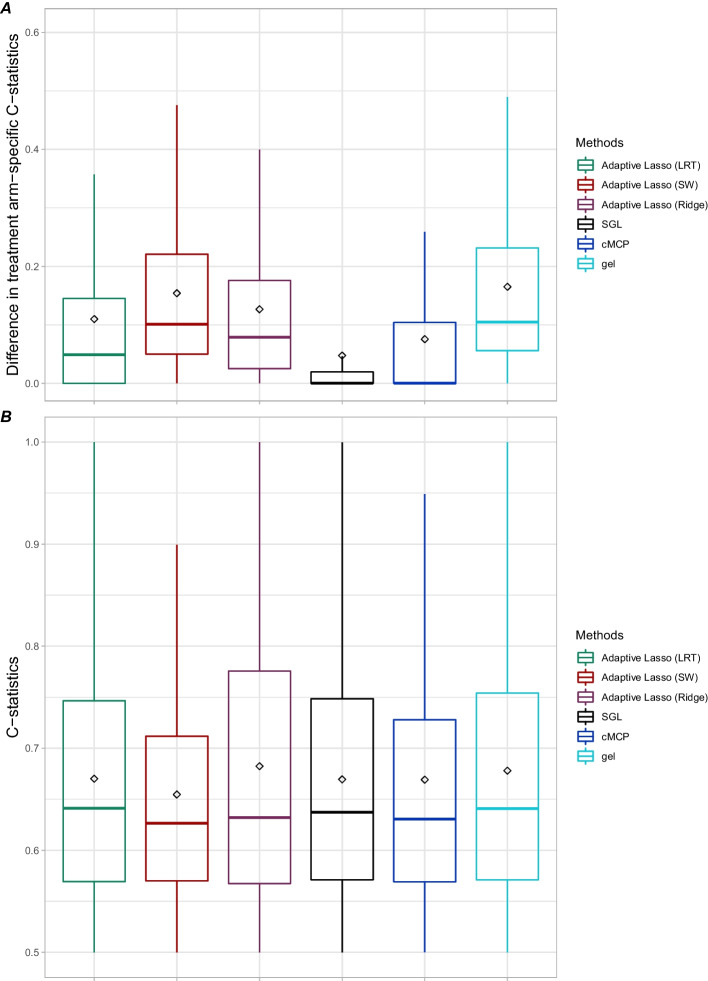
A: Box plots of absolute values of the difference ($\Delta$) in Uno C-statistic between the two treatment arms calculated on the 500 replications of the validation set (representing 40% of randomized clinical trial of breast cancer). B: Box plots of the Uno C-statistic calculated on the 500 replications of the validation set (representing 40% of randomized clinical trial of breast cancer). The box delineates the interquartile range (IQR) and contains a horizontal line corresponding to the median; outside the box, Tukey-style whiskers extend to a maximum of 1.5*IQR beyond the box. The black diamonds represent the average of the absolute values of the $\Delta$C-statistic (panel A) and the average of the C-statistic (panel B)

In terms of the concordance index, the strength of the selected interactions is globally not very high since the maximum of the mean of the Uno $\Delta$C statistic is about 0.16 (Fig. 5A). This is reached with the adaptive lasso (SW) and gel methods. These two methods correspond to the approaches that most favor the hierarchical constraint for at least one gene when compared with the other competitors. The gel method selected interactions with relative high regression coefficients, whereas the adaptive lasso (SW) method selected 10 times more gene-treatment interactions but with lower regression coefficients (Additional file 1: Fig. S3). To a lesser degree, the adaptive lasso (ridge) and the adaptive lasso (LRT) methods had an average of $\Delta$C-statistic equal to 0.13 and 0.11, respectively. These two methods have $\Delta$C-statistic values close to those of the adaptive lasso (SW) method, except that the adaptive lasso (LRT) method selects fewer interactions than the last two. This explains the lower median and mean $\Delta$C-statistic than these other two methods. As expected cMCP and SGL methods had a mean $\Delta$C-statistic close to 0 (0.07 and 0.05, respectively). While the former selected the lowest number of gene-processing interactions, the latter selected the lowest regression coefficients of interactions (close to 0). Figure 5B shows the distribution of the predictive performance (C-statistic) of the selected models by the different approaches on the validation sets. The two adaptive lasso approaches (SW and ridge) which identified the largest number of gene-treatment interaction (Fig. 4) have numerically similar C-statistic average compared to other approaches.

## Discussion

In this study, we proposed two weighting schemes for the adaptive lasso method for time-to-event outcomes to favor the biomarker-treatment interaction hierarchical constraint. For this purpose, we created groups composed of the biomarker main effect and its interaction with the treatment, and then performed a two-level selection of the groups and within the groups while favoring the hierarchical interaction constraint. The first weight was defined by the inverse of the Wald statistic of interaction and is identical for each component of the group. The second extension used a weight defined by the partial likelihood ratio test inversely proportional to the strength of the interaction between biomarker and treatment. These two adaptive lasso methods were compared through a simulated study to the adaptive lasso (ridge), cMCP, gel and SGL methods in terms of selection performance and strength interactions. Overall, the results indicated that the two proposed adaptive lasso methods (Single Wald and Likelihood ratio test) and gel favor more the hierarchical constraint of biomarker-treatment interaction compared to the other methods. They are characterized by a higher ratio between the number of selected main effects of the biomarkers and the number of its selected interactions. They yield the lowest FNR for main effects under all scenarios, but may entail a high FDR of selected interactions compared to other methods i.e. a high probability to select interactions which are noninfluent. This is the case, except for gel method, for situations where only one true predictive marker exist (possible situation in practise, scenario 2) and for situation where several predictive and prognostic biomarkers exist (less probable situation in practise, scenario 4 and 4b). All methods were consistent in selecting the true interactions but had more difficulties when prognostic biomarkers exist (scenario 4 and 4b). This is shown by the decrease of the difference of the Uno C-statistic between the two treatment arms from scenario without prognostic biomarkers (scenario 3) to scenario with 10 prognostic biomarkers (scenario 4). Finally, the general findings of the Wald weighting-based adaptive lasso were also found through the analysis of a randomised clinical trial in breast cancer patients. A high number of interactions were selected favoring the selection of its main effects but the robustness analysis with different cut-offs in the selection procedure suggested that some of gene-treatment interactions are probably false.

The idea of selecting more or less biomarker main effect when its interaction is selected in high-dimensional data is not new. Bien et al. [8] addressed this point in 2013 for Gaussian and binary outcomes and recently in Du et al. [16] investigated this topic in the context of RCTs. These authors proposed to use a more restrictive condition on the selection of interactions and corresponding main effects since they automatically force the latter when the former is selected. In addition, there are other parsimonious approaches such as [48, 49] that model important variables that interact with the treatment but without the constraint of hierarchical interaction.

In our work related to high-dimensional data analysis, we focused a priori on methods that favor the biomarker-treatment interaction hierarchy without necessarily forcing the main effect to be included in the final model in order to find a compromise between general parameter sparsity and the hierarchical constraint. An interesting extension of this work for low to middle-dimensional data problems would therefore consist of comparing the proposed approaches with approaches that force the hierarchical constraint [16] in simulated situations where hierarchical and no hierarchical structure is satisfied. In the setting of a randomized trial, however, biomarkers with interaction effects should often, but not always, have prognostic effects [50]. Despite the inherent limitations of simulations due to their arbitrary nature in the choice of parameters and scenarios, some recommendations can be drawn from our results. When we expect a low number of predictive biomarkers, the adaptive lasso with Single Wald weighting may be of interest. Otherwise, if we expect both a high number of predictive and prognostic biomarkers, the adaptive lasso with likelihood ratio test weighting seems the most appropriate approach. Although this article focuses on censored time-to-event outcomes and the Cox semi-parametric model, the approaches presented can be applied more generally to other types of outcomes (such as binary or continuous) and other models (as logistic or linear models). As perspectives of further work research, it could be also interesting to investigate the consideration of predictive pathways (i.e. group of biomarkers interacting with the treatment) in the selection procedure combined with the hierarchical constraint. A possible approach is to consider weights for the adaptive lasso method that take into account both the pathway information and the individual biomarker information. For the sake of simplicity, we assumed a log-linear relationship between the biomarkers and the risk of the occurrence of the outcome. Incorporating the generalized additive model selection [51, 52], which allows a greater flexibility by fitting sparse generalized additive models in high-dimension with *l*1 penalty, could generalize the methods that we investigated to overcome the log-linear assumption. To control the false positive rate of the proposed approaches in this paper, we could adapt the approach of Wang et al. [50] to the hierarchical constraint setting. This approach was developed to identify biomarker-treatment interactions in randomized clinical trials with control of familywise error rate. The authors used a screening procedure employing two independent stages: a stage 1 for screening biomarkers and a stage 2 to test treatment interaction on the biomarkers that passed the screening.

In conclusion, we proposed specific weightings with the adaptive lasso for addressing the recent question of hierarchical constraint of interaction in high dimensional data for censored outcomes. These approaches may be of particular interest for the research of putative biomarkers-treatment interactions which is more and more investigated in randomized clinical trials.

## Supplementary Information

**Additional file 1**. Supplementary information.

## Data Availability

The program code for the proposed approaches is available from the corresponding author, S.M. upon request.

## Abbreviations

Lasso: least absolute shrinkage and selection operator
AL: adaptive lasso
SW: Single Wald
LRT: likelihood ratio test
cMCP: composite Minimax Concave Penalty
gel: group exponential lasso
SGL: Sparse Group Lasso
FP: False positives
TP: true positives
FDR: False Discovery Rate
FNR: False Negatives Rate
$q_{Pe}$: number of predictive biomarkers
$q_{Po}$: number of prognostic biomarkers
$n_{Pe}$: number of selected interactions
$n_{Pe}$: number of selected main effects corresponding to the selected interactions
*HR*: Hazard Ratio
*Ctrl*: control arm
*Exp*: experimental arm

## References

[1] Le Tourneau C, Kamal M, Bièche I. Precision medicine in oncology: what is it exactly and where are we? Pers. Med. 2018;15(5):351–353. 10.2217/pme-2018-0036. arxiv: 3026.0312 10.2217/pme-2018-0036 30260312

[2] Stendahl M, Rydén L, Nordenskjöld B, Jönsson PE, Landberg G, Jirström K. High progesterone receptor expression correlates to the effect of adjuvant tamoxifen in premenopausal breast cancer patients. Clin Cancer Res. 2006;12(15):4614–8. 10.1158/1078-0432.CCR-06-0248 16899609

[3] Delozier T (2010). Hormonothérapie du cancer du sein. Journal de gynécologie obstétrique et biologie de la reproduction.

[4] Royston P, Sauerbrei W (2008). Interactions between treatment and continuous covariates: a step toward individualizing therapy. J Clin Oncol.

[5] Michiels S, Koscielny S, Hill C (2007). Interpretation of microarray data in cancer. Br J Cancer.

[6] Cox DR. Interaction. International Statistical Review/Revue Internationale de Statistique, 1984;1–24. 10.2307/1403235

[7] McCullagh P. Generalized linear models 2019.

[8] Bien J, Taylor J, Tibshirani R (2013). A lasso for hierarchical interactions. Ann Stat.

[9] Cox DR (1972). Regression models and life-tables. J R Stat Soc Ser B (Methodological).

[10] Tibshirani R (1996). Regression shrinkage and selection via the lasso. J R Stat Soc Ser B (Methodological).

[11] Zou H (2006). The adaptive lasso and its oracle properties. J Am Stat Assoc.

[12] Simon N, Friedman J, Hastie T, Tibshirani R (2013). A sparse-group lasso. J Comput Gr Stat.

[13] Yuan M, Lin Y (2006). Model selection and estimation in regression with grouped variables. J R Stat Soc Ser B (Methodological).

[14] Hastie T. Statistical Learning with Sparsity:The Lasso and Generalizations. Taylor & Francis, Andover, England 2015. 10.1201/b18401

[15] Ternes N, Rotolo F, Heinze G, Michiels S (2017). Identification of biomarker-by-treatment interactions in randomized clinical trials with survival outcomes and high-dimensional spaces. Biom J.

[16] Du Y, Chen H, Varadhan R (2021). Lasso estimation of hierarchical interactions for analyzing heterogeneity of treatment effect. Stat Med.

[17] Chipman H (1996). Bayesian variable selection with related predictors. Can J Stat.

[18] Hamada M, Wu CJ (1992). Analysis of designed experiments with complex aliasing. J Qual Technol.

[19] Nelder J (1977). A reformulation of linear models. J R Stat Soc Ser A (General).

[20] Zhang HH, Lu W (2007). Adaptive lasso for cox’s proportional hazards model. Biometrika.

[21] Belhechmi S, De Bin R, Rotolo F, Michiels S (2020). Accounting for grouped predictor variables or pathways in high-dimensional penalized cox regression models. BMC Bioinform.

[22] Hoerl AE, Kennard RW (1970). Ridge regression: biased estimation for nonorthogonal problems. Technometrics.

[23] Zou H, Hastie T (2005). Regularization and variable selection via the elastic net. J R Stat Soc Ser B (Stat Methodol).

[24] Breheny P, Huang J (2009). Penalized methods for bi-level variable selection. Stat Interface.

[25] Huang J, Breheny P, Ma S. A selective review of group selection in high-dimensional models. Stat Sci Rev J Inst Math Stat 2012;27(4). 10.1214/12-STS392 10.1214/12-STS392 PMC3810358 24174707

[26] Zhang C-H (2010). Nearly unbiased variable selection under minimax concave penalty. Ann Stat.

[27] Breheny P (2015). The group exponential lasso for bi-level variable selection. Biometrics.

[28] Verweij PJ, Van Houwelingen HC (1993). Cross-validation in survival analysis. Stat Med.

[29] Verweij PJ, Van Houwelingen HC (1994). Penalized likelihood in cox regression. Stat Med.

[30] Genovese C, Wasserman L (2002). Operating characteristics and extensions of the false discovery rate procedure. J R Stat Soc Ser B (Stat Methodol).

[31] Pawitan Y, Michiels S, Koscielny S, Gusnanto A, Ploner A (2005). False discovery rate, sensitivity and sample size for microarray studies. Bioinformatics.

[32] Michiels S, Potthoff RF, George SL (2011). Multiple testing of treatment-effect-modifying biomarkers in a randomized clinical trial with a survival endpoint. Stat Med.

[33] Schemper M (1988). Non-parametric analysis of treatment-covariate interaction in the presence of censoring. Stat Med.

[34] Uno H, Cai T, Pencina MJ, D’Agostino RB, Wei L-J (2011). On the c-statistics for evaluating overall adequacy of risk prediction procedures with censored survival data. Stat Med.

[35] Ternes N, Rotolo F, Michiels S. Biospear: Biomarker Selection in Penalized Regression Models. 2017. R package version 1.0.1. https://CRAN.R-project.org/package=biospear 10.1093/bioinformatics/btx560 28927242

[36] Ternes N, Rotolo F, Michiels S (2018). biospear: an r package for biomarker selection in penalized cox regression. Bioinformatics.

[37] Simon N, Friedman J, Hastie T, Tibshirani R. Regularization paths for cox’s proportional hazards model via coordinate descent. J Stat Softw 2011;39(5):1. 10.18637/jss.v039.i05 10.18637/jss.v039.i05 PMC4824408 27065756

[38] Friedman J, Hastie T, Simon N, Tibshirani R. Glmnet: Lasso and Elastic-Net Regularized Generalized Linear Models. 2018. R-package version 2.0-16. https://cran.r-project.org/web/packages/glmnet

[39] Patrick B. Grpreg: Regularization Paths for Regression Models with Grouped Covariates. 2020. R package version 3.3.0. https://CRAN.R-project.org/package=grpreg

[40] Noah S, Jerome F, Trevor H, Rob T. SGL: Fit a GLM (or Cox Model) with a Combination of Lasso and Group Lasso Regularization. 2019. R package version 1.3. https://CRAN.R-project.org/package=SGL

[41] L P-GK, Chungyeul K, Jong-Hyeon J, Noriko T, Hanna B, G GP, Debora F, C GL, Nour S, Eike B *et al.* Predicting degree of benefit from adjuvant trastuzumab in nsabp trial b-31. J Natl Cancer Inst 2013;105(23):1782–1788. 10.1093/jnci/djt321 10.1093/jnci/djt321 PMC3848987 24262440

[42] Roberts S, Nowak G (2014). Stabilizing the lasso against cross-validation variability. Comput Stat Data Anal.

[43] Miyake M, Nakano K, Itoi S-I, Koh T, Taki T (1996). Motility-related protein-1 (mrp-1/cd9) reduction as a factor of poor prognosis in breast cancer. Cancer Res.

[44] Huang C-L, Kohno N, Ogawa E, Adachi M, Taki T, Miyake M (1998). Correlation of reduction in mrp-1/cd9 and kai1/cd82 expression with recurrences in breast cancer patients. Am J Pathol.

[45] Koh HM, Jang BG, Lee DH, Hyun CL (2021). Increased cd9 expression predicts favorable prognosis in human cancers: a systematic review and meta-analysis. Cancer Cell Int.

[46] Jansen MP, Ruigrok-Ritstier K, Dorssers LC, van Staveren IL, Look MP, Meijer-van Gelder ME, Sieuwerts AM, Helleman J, Sleijfer S, Klijn JG (2009). Downregulation of siah2, an ubiquitin e3 ligase, is associated with resistance to endocrine therapy in breast cancer. Breast Cancer Res Treat.

[47] Chan P, Möller A, Liu MC, Sceneay JE, Wong CS, Waddell N, Huang KT, Dobrovic A, Millar EK, O’Toole SA (2011). The expression of the ubiquitin ligase siah2 (seven in absentia homolog 2) is mediated through gene copy number in breast cancer and is associated with a basal-like phenotype and p53 expression. Breast Cancer Res.

[48] Tian L, Alizadeh AA, Gentles AJ, Tibshirani R (2014). A simple method for estimating interactions between a treatment and a large number of covariates. J Am Stat Assoc.

[49] Lu W, Zhang HH, Zeng D (2013). Variable selection for optimal treatment decision. Stat Methods Med Res.

[50] Wang J, Patel A, Wason JM, Newcombe PJ (2022). Two-stage penalized regression screening to detect biomarker-treatment interactions in randomized clinical trials. Biometrics.

[51] Chouldechova A, Hastie T. Generalized additive model selection. arXiv preprint arXiv:1506.03850 2015.

[52] Hastie TJ, Tibshirani RJ. Generalized Additive Models. Routledge, New York 2017. 10.1201/9780203753781

