# Supplementary material for: Favoring the hierarchical constraint in penalized survival models for randomized trials in precision medicine
Source: BMC Bioinformatics. 2023 Mar 16;24:96. doi: 10.1186/s12859-023-05162-x (PMC10022294; doi:10.1186/s12859-023-05162-x)

Appendix

Figure S1 A: The difference in arm-specific Uno C-statistics (ΔC-statistics) between the training and validation sets in the alternative scenarios. The different symbols represent the average of the estimated ΔC-statistics value in the validation sets. The vertical lines represent the reduction in ΔC-statistics from the training set to the validation set. B: Box plots of the Uno C-statistic calculated on the 500 replications of the training set and of the validation set. The box delineates the interquartile range (IQR) and contains a horizontal line corresponding to the median; outside the box, Tukey-style whiskers extend to a maximum of 1.5*IQR beyond the box. The black diamonds represent the average of the absolute values of the ΔC-statistic (panel A) and the average of the C-statistic (panel B).

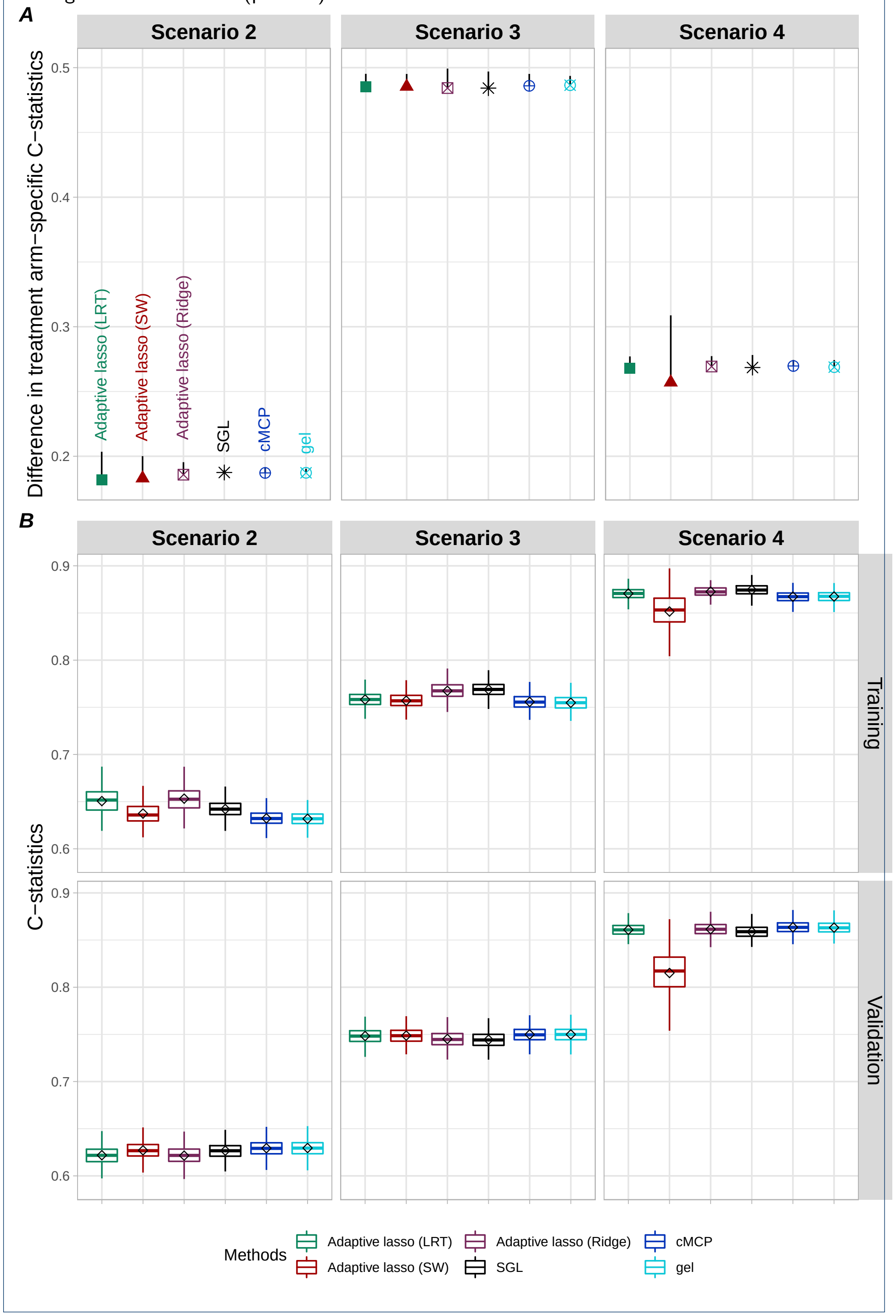

Table S1 Selection performance of biomarker-treatment interactions and main effects corresponding to the selected interactions for scenario 4b with $n = 1500$ patients and $p = 5000$ biomarkers.

			AL(LRT)	AL(SW)	AL(ridge)	SGL	cMCP	gel
Scenario 4b	Interactions	n_{Pe}	15.94	106.81	5.27	11.81	16.34	19.49
$q_{Pe} = 10$		TP / FP	9.95/5.99	9.94/96.88	0.97/4.30	10.00/1.81	10.00/6.34	9.99/9.49
$q_{Po} = 10$		FDR / FNR	0.35/0.00	0.90/0.01	0.58/0.903	0.14/0.00	0.36/0.00	0.49/0.00
	Main effects	n_{Po} (n_{Po}/n_{Pe})	6.42 (40%)	49.28 (46%)	0.80 (15%)	0.74 (6%)	1.49 (9%)	13.91 (71%)
		TP / FP	4.42/2.00	7.04/42.24	0.11/0.68	0.57/0.16	0.50/0.99	4.65/9.26
		FDR / FNR	0.29/0.56	0.85/0.30	0.35/0.99	0.12/0.94	0.52/0.95	0.68/0.54

q_{Pe} number of predictive biomarkers, q_{Po} number of prognostic biomarkers, n_{Pe} number of selected interactions, n_{Po} number of selected main effects corresponding to the selected interactions, FP false positives, TP true positives, FDR false discovery rate, FNR false negative rate, AL adaptive lasso, LRT likelihood ratio test, SW single Wald.

Figure S2 A: The difference in arm-specific Uno C-statistics (ΔC-statistics) between the training and validation sets in the scenario 4b (extension of the scenario 4 in changing the biomarkers number from $p = 500$ to 5000). The different symbols represent the average of the estimated ΔC-statistics value in the validation sets. The vertical lines represent the reduction in ΔC-statistics from the training set to the validation set. B: Box plots of the Uno C-statistic calculated on the 500 replications of the training set and of the validation set. The box delineates the interquartile range (IQR) and contains a horizontal line corresponding to the median; outside the box, Tukey-style whiskers extend to a maximum of 1.5*IQR beyond the box. The black diamonds represent the average of the absolute values of the ΔC-statistic (panel A) and the average of the C-statistic (panel B)

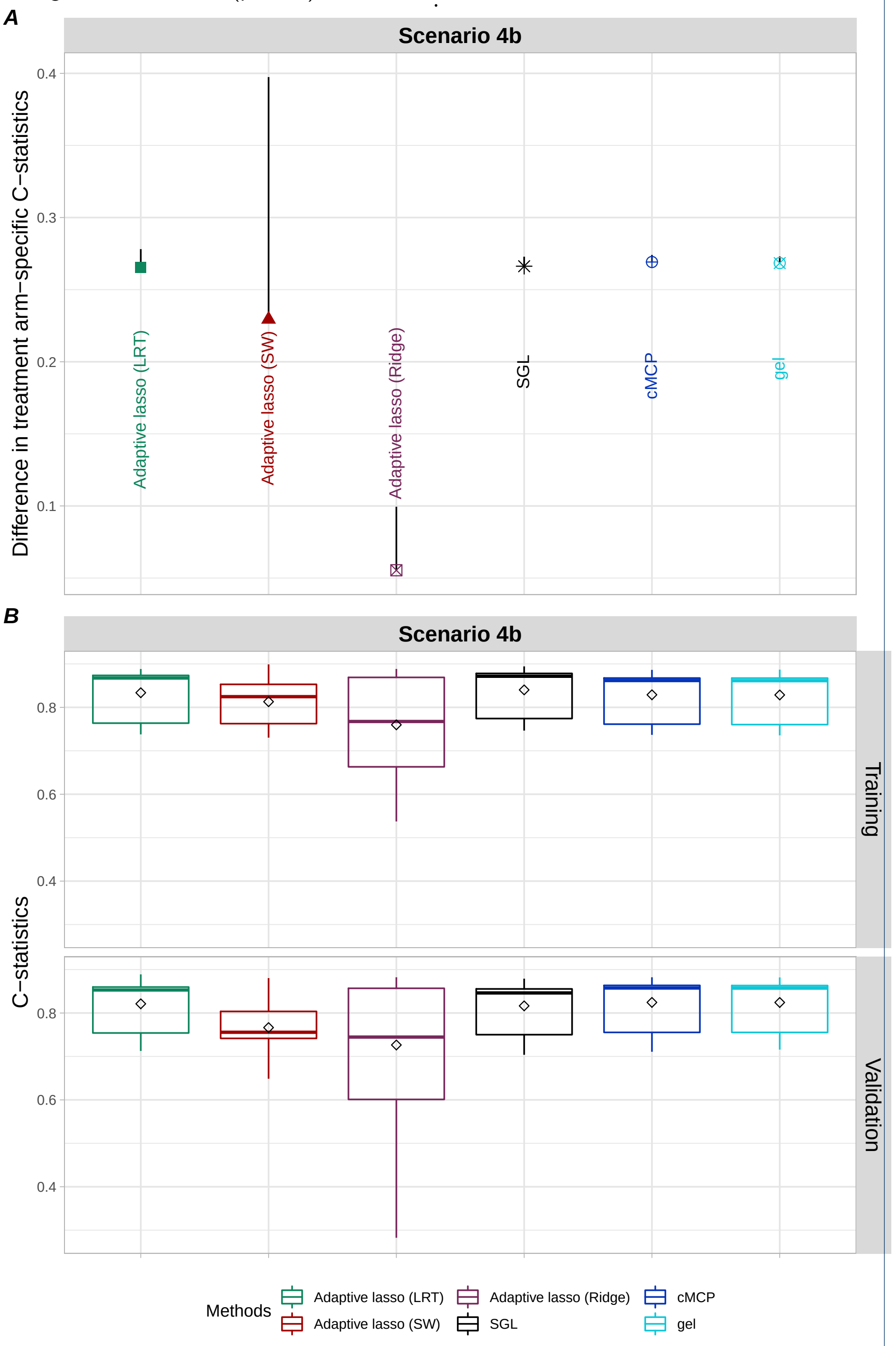

Figure S3 Scatter plot of regression coefficients for gene-treatment interactions selected across 500 replicates. The number of gene-treatment interactions selected across these replications is 1512, 5618, 4524, 270, 262, and 550 for AL(RT), AL(SW), AL (Ridge), SGL, cMCP, and gel methods, respectively.

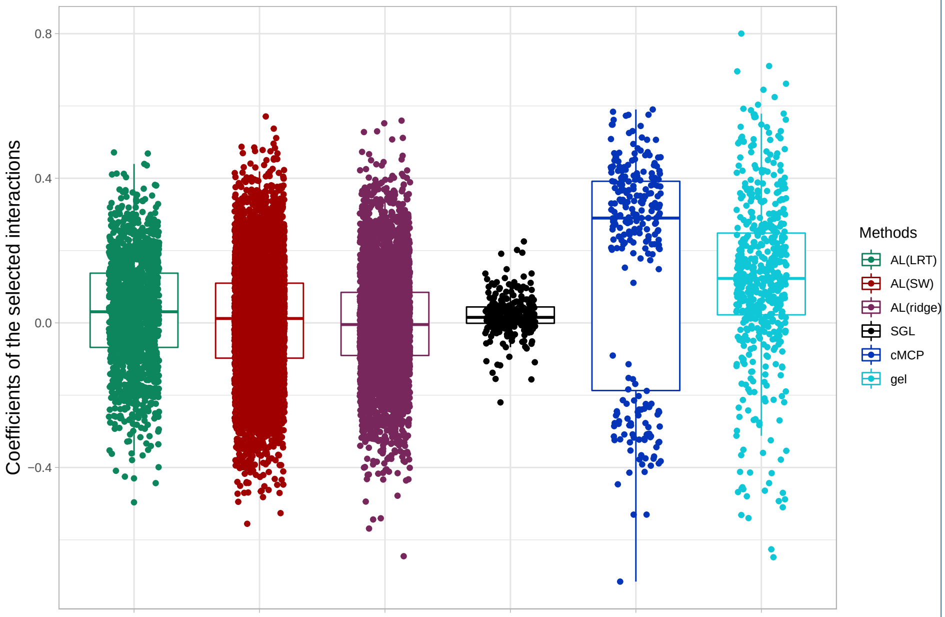

Supplement: Supplementary file 1 — Additional file 1. Supplementary information. [file 12859_2023_5162_MOESM1_ESM.pdf]
